# On-surface synthesis and characterization of polyynic carbon chains

**DOI:** 10.1093/nsr/nwae031

**Published:** 2024-01-22

**Authors:** Wenze Gao, Wei Zheng, Luye Sun, Faming Kang, Zheng Zhou, Wei Xu

**Affiliations:** Interdisciplinary Materials Research Center, School of Materials Science and Engineering, Tongji University, Shanghai 201804, China; Interdisciplinary Materials Research Center, School of Materials Science and Engineering, Tongji University, Shanghai 201804, China; Interdisciplinary Materials Research Center, School of Materials Science and Engineering, Tongji University, Shanghai 201804, China; Interdisciplinary Materials Research Center, School of Materials Science and Engineering, Tongji University, Shanghai 201804, China; Interdisciplinary Materials Research Center, School of Materials Science and Engineering, Tongji University, Shanghai 201804, China; Interdisciplinary Materials Research Center, School of Materials Science and Engineering, Tongji University, Shanghai 201804, China

**Keywords:** polyyne, scanning probe microscopy, carbon allotrope, on-surface synthesis, organometallic polyyne

## Abstract

Carbyne, an elusive *sp*-hybridized linear carbon allotrope, has fascinated chemists and physicists for decades. Due to its high chemical reactivity and extreme instability, carbyne was much less explored in contrast to the *sp^2^*-hybridized carbon allotropes such as graphene. Herein, we report the on-surface synthesis of polyynic carbon chains by demetallization of organometallic polyynes on the Au(111) surface; the longest one observed consists of ∼60 alkyne units (120 carbon atoms). The polyynic structure of carbon chains with alternating triple and single bonds was unambiguously revealed by bond-resolved atomic force microscopy. Moreover, an atomically precise polyyne, C_14_, was successfully produced via tip-induced dehalogenation and ring-opening of the decachloroanthracene molecule (C_14_Cl_10_) on a bilayer NaCl/Au(111) surface at 4.7 K, and a band gap of 5.8 eV was measured by scanning tunnelling spectroscopy, in a good agreement with the theoretical HOMO–LUMO gap (5.48 eV).

## Teaser

A carbon chain with ∼120 atoms has been synthesized on the surface and characterized by bond-resolved scanning probe microscopy.

## INTRODUCTION

The creation of new carbon forms with different topologies has emerged as an exciting topic in both fundamental chemistry and materials science. Since the first discovery of fullerenes [1], carbon nanotubes (CNTs) [2,3], and graphene [4], the family of *sp*^2^ carbon allotropes have been broadly explored and investigated which exhibit unusual chemical and physical properties [5]. Some recent studies have enriched this family to seek new polymeric carbon forms, such as fullerene networks [6,7]. In contrast, carbyne, an *sp*-hybridized carbon allotrope with an infinite one-dimensional (1D) carbon chain attracted more interest back in the 1960s, but its structure remained controversial over the decades [8,9]. Unlike conventional 1D carbon allotrope CNTs, carbyne is a real ultimate 1D structure, which has a cross-sectional dimension reduced to only one carbon atom. Carbyne owns the potential to be the mechanically strongest known material due to its unique structure [10]. However, the great challenge of synthesizing accessible carbyne, caused by its high chemical reactivity and extreme instability, impeded the experimental confirmation and further exploration of its applications.

**Figure 1.**
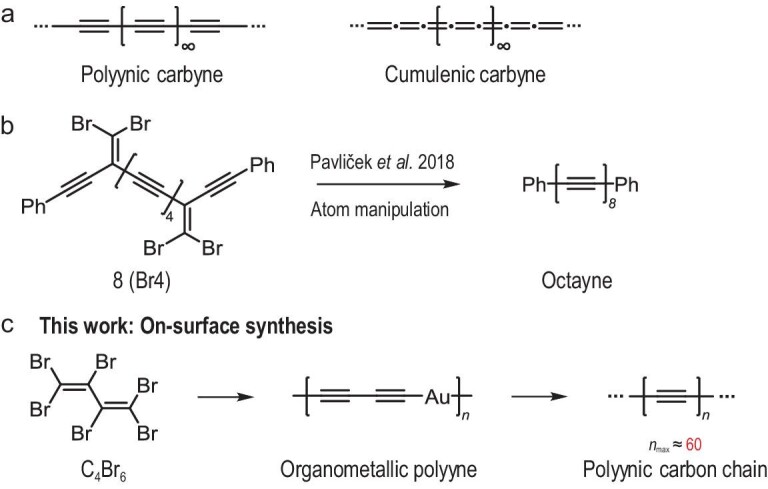
Synthetic strategies toward the polyynic carbon chain (PCC). (a) Two possible structures of carbyne. (b) Previous work towards the synthesis of polyyne compounds (with 8 alkyne units) by atomic manipulation on NaCl. (c) In this work, an on-surface synthesis strategy is applied where the C_4_Br_6_ precursor is employed to form PCC with the longest one observed consisting of ∼60 alkyne units.

In principle, carbyne can be either polyynic with an alternation of single and triple bonds, or cumulenic with a connection of consecutive double bonds (Fig. 1a), while the former has been proven to be energetically more favorable [11]. Thus, the experimental attempts in this field have focused heavily on the synthesis of polyynes by different methods to model carbyne. In order to stabilize long carbon chains, adding endgroups is a common organic synthetic strategy for the preparation of polyyne with different lengths [12–17], and the longest polyyne reported so far consists of 24 contiguous alkyne units (48 carbons) [17]. However, longer polyynes commonly require heavier endgroups for stabilization, limiting the production of long polyynic carbon chains. Instead, single- and double-walled CNTs, known as confining nanoreactors and protectors to produce and stabilize highly active structures, have been used to prepare long carbon chains comprising more than 6000 carbon atoms [18].

**Figure 2.**
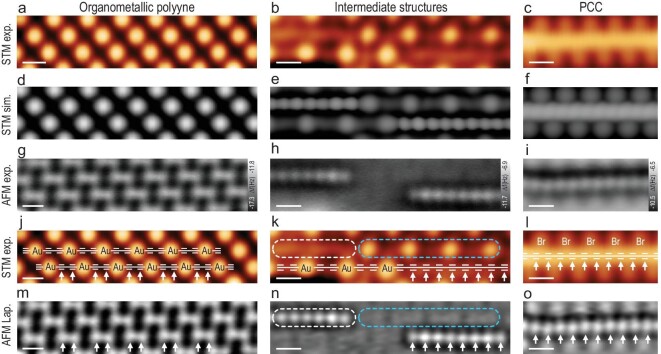
Formation of polyynic carbon chain by demetallization of organometallic polyyne. STM images (a–c, j–l), simulated STM images (d–f), AFM images (g–i), and Laplace-filtered AFM images (m–o) of organometallic polyynes, intermediate structures, and polyynic carbon chain, respectively. The STM images in (j–l) are overlaid with the chemical structures as guides. In the intermediate structure (k, n), the partially demetallized and the remaining organometallic polyyne are outlined by white and blue contours, respectively. The alkyne units are indicated by white arrows in (j–o). All STM images are taken at *V* = 0.40 V, *I*_t_ = 100 pA; all AFM images are recorded with a Br-functionalized tip at different tip offsets Δ*z* (g: Δ*z* = −370 pm, h: Δ*z* = −130 pm, i: Δ*z* = −260 pm) with respect to an STM set point (*V* = 0.40 V, *I*_t_ = 100 pA). Scale bars: 0.5 nm.

**Figure 3.**
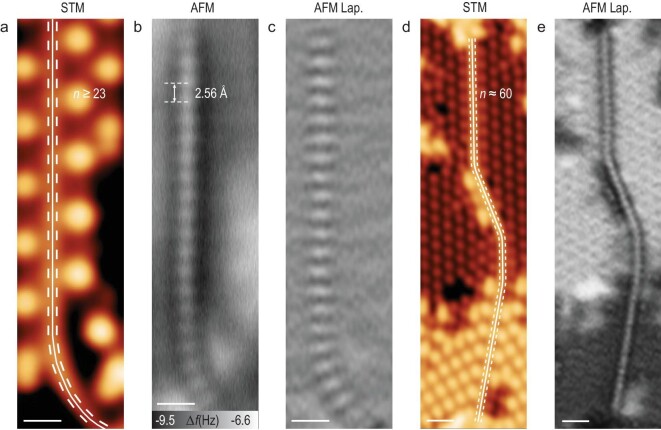
Real-space imaging of long polyynic carbon chains. (a–e) STM images (a, d), AFM images (b), and Laplace-filtered AFM images (c, e) of polyynic carbon chains with at least 23 (a–c) and ∼60 (d, e) alkyne units, respectively. The STM images in (a) and (d) are overlaid with the chemical structure as guides. The STM images are all taken at: *V* = 0.40 V, *I*_t_ = 100 pA. The AFM images are recorded with a Br-functionalized tip at different tip offsets Δ*z* (b: Δz = −75 pm, e: Δz = −80 pm) with respect to an STM set point (*V* = 0.40 V, *I*_t_ = 100 pA). Scale bars: 0.5 nm (a–c); 1 nm (d–e).

**Figure 4.**
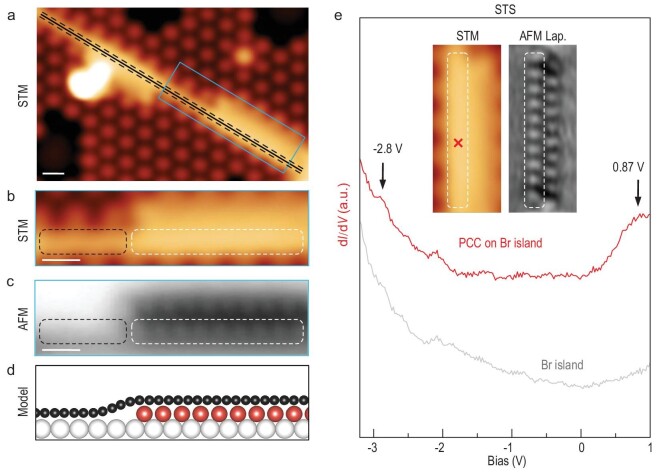
Characterization of the electronic properties of partially decoupled polyynic carbon chains. (a) An STM image of a PCC which is partially decoupled by Br atoms. A chemical structure is overlaid as a guide. (b–d) The STM image (b), AFM image (c), and schematic model (d) of partially decoupled PCC outlined by a blue contour in (a). The PCC segments on Br island and on Au(111) are indicated by white and black contours, respectively, in (b, c). Black, red, and white balls represent C, Br, and Au atoms, respectively. (e) The d*I*/d*V* spectrum of the PCC segment on Br island acquired at the position indicated by the red cross in the inserted STM image (red curve) and the reference spectrum taken on the Br island (grey curve). The spectra are vertically shifted for clarity. All the STM images are taken at *V* = 0.40 V, *I*_t_ = 100 pA. The AFM image (c) is recorded with a Br-functionalized tip at tip offset Δ*z* = −160 pm with respect to an STM set point (*V* = 0.40 V, *I*_t_ = 100 pA). Scale bars: 0.5 nm.

On-surface synthesis is emerging as a promising approach for atomically precise fabrication of highly reactive 1D nanostructures with *sp*-hybridization that could be hardly synthesized via conventional solution synthetic chemistry [19–22]. Developments in scanning tunneling microscopy (STM) and atomic force microscopy (AFM) have enabled the designing and *in-situ* characterization of low-dimensional carbon nanostructures with unprecedented resolution on the atomic scale and chemical-bond level [23–25]. Benefiting from the development of scanning probe microscopies above, a series of polyynes comprising 3, 4, 6, and 8 alkyne units have been successfully generated via skeletal rearrangement induced by atomic manipulation on NaCl at 5 K (Fig. 1b), and the polyynic structure was well-revealed by AFM [26]. However, its length is still limited by endgroups. Herein, we show how an on-surface synthesis approach can be developed to synthesize polyynic carbon chains (PCCs) without endgroups, and the longest one consists of ∼60 alkyne units. As shown in Fig. 1c, 1,1,2,3,4,4-hexabromobutadiene (C_4_Br_6_) molecules were first polymerized through debrominative coupling on the Au(111) surface followed by the formation of organometallic polyynes [20], and in the second step, these organometallic polyynes underwent demetallization by further annealing to form PCCs. A low-temperature STM/AFM was used to reveal the polyynic structure of carbon chains with bond-resolved resolution. Moreover, a specific C_14_ polyyne was also successfully produced via tip-induced dehalogenation and ring-opening of the decachloroanthracene molecule (C_14_Cl_10_) on a bilayer NaCl/Au(111) surface at 4.7 K. The electronic properties were further studied through a combination of scanning tunnelling spectroscopy (STS) and density functional theory (DFT) calculations, and a band gap of the C_14_ polyyne was measured to be 5.8 eV, in good agreement with the theoretical HOMO–LUMO gap (5.48 eV). Our results provide a feasible route for the synthesis of long and stable polyynic carbon chains by demetallization of organometallic polyynes on Au(111), and the on-surface generation of a carbon chain with a specific length on NaCl/Au(111) would be potentially useful for investigating the intrinsic properties of linear carbons.

## RESULTS AND DISCUSSION

### Synthesis and structural characterization

The C_4_Br_6_ was synthesized in solution through a two-step sequence (see Methods and Fig. S1 for synthetic details and NMR spectra) and then deposited onto a clean Au(111) surface held at 300 K in an ultrahigh vacuum, resulting in the formation of diacetylenic organometallic polyynes (**−**C≡C**−**C≡C**−**Au**−**) (Fig. 2, column 1) through debrominative organometallic coupling, which is similar to our previously reported work [20]. The bright protrusions in the STM image (Fig. 2a) resulted from the electronic density of states of Au atoms, as illustrated by the overlaid chemical structure in Fig. 2j. From the AFM images in Fig. 2g and m, the diyne moieties are clearly resolved as two discrete characteristic protrusions indicated by white arrows [25]. The distance between two C–C triple bonds is measured to be 2.62 Å (Fig. S2d).

The organometallic polyynes started to partially demetallize [27] as the sample was annealed to 380 K for 60 min, resulting in the formation of short PCCs within the organometallic polyynes (Fig. 2, column 2), in which the short PCC and the organometallic polyyne are outlined by white and blue contours in Fig. 2k, n, respectively. The bond-resolved AFM contrast provided conclusive evidence for a polyynic structure of PCCs with defined positions of triple bonds as indicated by white arrows in Fig. 2n with reference to the chemical structure overlaid in Fig. 2k [26]. The periodicity of PCCs is measured to be 2.56 Å (Fig. S2e) which corresponds well with the theoretical value (Fig. S3) and could be clearly distinguished from that of the acetylenic organometallic polyynes (5.20 Å) (Fig. S2c, f). Notably, within intermediate structures (Fig. 2, column 2), organometallic polyyne parts are not distinguishable in the AFM images at the imaging height of PCCs (Fig. 2n). This should be attributed to the lower adsorption height of the organometallic polyyne due to stronger interaction with the substrate as compared to the PCC (Fig. S3). The PCCs became longer during further continuous annealing at 380 K for 120 min (Fig. 2, column 3). The AFM images (Fig. 2i, o) revealed a polyynic structure of PCC with a length of 12 alkyne units as illustrated by the chemical structure in Fig. 2l. The assignment of all products was further supported by STM simulations (Fig. 2d–f).

Longer PCCs with a length of at least 23 alkyne units (46 carbon atoms) were also observed (Fig. 3a), in which the defined positions of 23 triple bonds are nicely revealed by AFM imaging (Fig. 3b, c). The organometallic polyynes on both sides of PCC are not distinguishable in AFM images at the typical imaging height of PCC, which is in agreement with the situation in Fig. 2n. Significantly, the longest PCC we observed consists of ∼60 alkyne units (120 carbon atoms) based on its length (∼15 nm), as shown in Fig. 3d, e. It can be seen from the Laplace-filtered AFM image (Fig. 3e) that the structure of this PCC is still intact without defects, and the polyynic nature (characteristic protrusions originated from C–C triple bonds) remains visible. STM images of other representative PCCs with lengths of ∼7, 10 and 12 nm are shown in Fig. S4.

### Electronic properties of PCCs

The electronic properties of PCCs were measured by STS. Due to the electronic coupling between the PCC and the metal surface, the detection of the electronic states was obscured and only the unoccupied state was detected at 1.17 V (Fig. S6). d*I*/d*V* maps acquired at this peak demonstrate that the corresponding state is highly localized at the PCC, without any characteristic feature (Fig. S6). More acutely, excessive Br adatoms resulting from the debromination of C_4_Br_6_ precursors affected the detection of the pure Au(111) surface states. As a result, a confined electronic state was detected at the centre of the pore surrounded by Br adatoms (Fig. S7) [28]. To obtain the intrinsic electronic properties of PCC, a partially decoupled one by intercalating Br atoms (indicated by the blue contour in Fig. 4a) was further characterized. The partially decoupled PCC segment exhibited a higher apparent height in the STM image (marked by the white contour in Fig. 4b) than the segment directly adsorbed on the metal substrate (marked by the black contour), as illustrated by the schematic model in Fig. 4d. This was further verified by the AFM image as the PCC segment on Au(111) was not imaged at the imaging height of the decoupled PCC segment (Fig. 4c). The close-up Laplace-filtered AFM image revealed that the decoupled PCC segment consists of 10 alkyne units (Fig. S8).

A d*I*/d*V* spectrum taken at the decoupled PCC segment is shown in Fig. 4e. In contrast to the results on Au(111), this spectrum exhibits two resonance peaks at −2.8 V and 0.87 V, respectively, which can be assigned to the occupied and unoccupied states, resulting in a band gap of approximately 3.67 eV. The characteristic nodes shown in the middle of every triple bond in the PCC segment in the constant height d*I*/d*V* mapping acquired at 0.87 V are in agreement with DFT-calculated LUMO (Fig. S9).

### C_14_ polyyne formation via atomic manipulation

**Figure 5.**
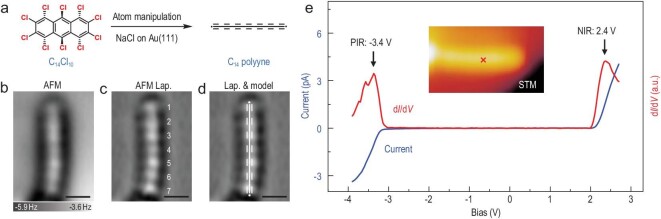
On-surface synthesis and characterization of a C_14_ polyyne via tip-induced dehalogenation and ring-opening of C_14_Cl_10_ on NaCl/Au(111). (a) Scheme of the formation of C_14_ polyyne. (b–d) AFM image (b) and Laplace-filtered AFM images (c, d) of a polyyne with 7 alkyne units. The chemical structure is overlaid in (d) as a guide. (e) The d*I*/d*V* spectrum of C_14_ polyyne (red curve) on NaCl/Au(111) acquired at the position indicated by the red cross in the inserted STM image along with the corresponding constant-height current spectrum (blue curve). AFM images were recorded with a CO-terminated tip at tip offset Δ*z* = −50 pm with respect to the STM set point (*I* = 1 pA, *V* = 0.3 V) above the NaCl surface. Scale bars: 0.5 nm.

To achieve a fully decoupled PCC and measure its intrinsic electronic property, another molecular precursor, decachloroanthracene (C_14_Cl_10_), was synthesized with the aim of generating a specific C_14_ carbon chain via tip-induced dehalogenation and ring-opening (Fig. 5a). C_14_Cl_10_ molecules were deposited onto a bilayer NaCl surface on Au(111) held at 6 K. To generate C_14_ polyyne, the tip was initially positioned on a single C_14_Cl_10_ molecule and retracted by about 4 Å from the STM set point (*I* = 3 pA, *V* = 0.3 V). Voltage pulses (4.0–4.5 V) were applied for a short duation (500 ms) at a constant tip height on the C_14_Cl_10_ molecule to induce full dehalogenation and accompanied ring opening. As a result, the polyynic C_14_ chain without endgroups was successfully generated as shown in the AFM images (Fig. 5b–d). According to the Laplace-filtered AFM image (Fig. 5c), seven characteristic protrusions can be clearly identified, attributed to seven C–C triple bonds as illustrated by the overlaid chemical structure in Fig. 5d, which could be unambiguously assigned to an individual C_14_ polyyne.

More importantly, because of decoupling by the insulating bilayer NaCl film, the intrinsic electronic property of C_14_ polyyne can be accurately characterized by STS with a CO-functionalized tip. Differential conductance spectrum (d*I*/d*V*) acquired on the C_14_ polyyne exhibits two pronounced peaks at –3.4 V and 2.4 V (Fig. 5e), corresponding to the positive and negative ion resonances (PIR and NIR), respectively, exhibiting a band gap of 5.8 eV, which is in a good agreement with the calculated HOMO–LUMO gap of the C_14_ polyyne (5.48 eV) at the ωB97XD/def2-TZVP level. Notably, the electronic properties of C_14_ polyyne can be significantly affected by the endgroups. Based on theoretical calculations, it is evident that the hydrogen-capped C_14_ polyyne possesses a larger HOMO–LUMO gap (6.86 eV) compared to the uncapped C_14_ polyyne with one radical at each end (5.48 eV) (Fig. S10). This finding also serves as evidence that the on-surface synthesized C_14_ polyyne was a pure carbon chain.

## CONCLUSION

In summary, we have synthesized a polyynic carbon chain consisting of ∼60 contiguous alkyne units by debrominative coupling of C_4_Br_6_ molecules and subsequent demetallization on the Au(111) surface. The polyynic structure of PCCs was well-revealed by bond-resolved AFM. Moreover, a specific C_14_ polyyne was also successfully synthesized via atomic manipulation on a NaCl/Au(111) surface at 4.7 K, and a band gap of 5.8 eV was measured by STS. Our results provide bond-resolved experimental insights into the structure of the polyynic carbon chains and open an avenue for the synthesis and characterization of long polyynes without endgroups.

## METHODS

### STM and AFM measurements

The experiments were carried out in a low-temperature STM/nc-AFM (CreaTec) under ultra-high vacuum conditions (base pressure below 1 × 10^−10^ mbar). Au(111) single crystals purchased from MaTeck were used as substrates for the growth of PCCs. Preparation of clean Au(111) surfaces was achieved by cycles of Ar^+^ ion sputtering and annealing at 850 K. The C_4_Br_6_ precursors were deposited (evaporator temperature 300 K) onto the clean Au(111) surface held at room temperature. After deposition, the sample was post-annealed to 380 K for 120 min to complete the reactions. C_14_Cl_10_ precursors were deposited (evaporator temperature 380 K) on cold NaCl/Au(111) surface held at 6 K. NaCl films were grown on Au(111) held at room temperature, resulting in islands of two and three monolayer thickness.

STM images were acquired in the constant-current mode at sample temperatures of 4.8 K. Non-contact AFM measurements were performed with a Pt/Ir tip attached to a tuning fork sensor. The tip was functionalized by controlled picking up of a CO molecule [24] (Fig. 5) or a Br atom [29] (other figures) at the tip apex from Au(111). CO molecules for tip modification were dosed onto the cold sample via a leak valve. We used a qPlus sensor [30] with a resonance frequency *f*_0_ = 29.49 kHz, quality factor *Q* ≈ 45 000 and a spring constant *k* ≈ 1800 N/m operated in frequency-modulation mode [31]. AFM images were acquired in constant-height mode at V = 0 *V* and an oscillation amplitude of *A* = 1 Å. The tip-height offsets Δ*z* for constant-height AFM images are defined as the offset in tip-sample distance relative to the STM set point at the Au(111) surface. The positive (negative) values of Δ*z* correspond to the tip-sample distance as increased (decreased) with respect to the STM set point.

The differential conductance (d*I*/d*V*) measurements were performed in the low-temperature STM/AFM at 4.8 K via the lock-in technique with a peak-to-peak bias-voltage modulation of 20 mV at a frequency of 768 Hz. The tips for d*I*/d*V* measurements are the same CO/Br-functionalized tips used for high-resolution AFM imaging. All the d*I*/d*V* maps were acquired in constant-height mode.

### Theoretical calculations

The structural optimizations of PCCs and organometallic polyynes on Au(111) were performed in the framework of DFT by using the Vienna *ab initio* simulation package (VASP) [32,33]. The projector-augmented wave method [34,35] was used to describe the interaction between ions and electrons, and the Perdew-Burke-Ernzerhof generalized gradient approximation (GGA) exchange-correlation functional was employed [36]. The dispersion-corrected DFT-D3 method [37] was used to consider the van der Waals interactions. The Tersoff-Hamann method [38] was used to obtain the simulated STM images. ωB97XD exchange-correlation functional [39] in conjunction with def2-TZVP [40] basis sets was used for the electronic HOMO–LUMO gaps of the C_14_ polyyne using the Gaussian 16 program package [41] combined with Multiwfn 3.8 code and Visual Molecular Dynamics (VMD 1.9) [42].

### Synthesis of precursors

1,1,2,3,4,4-Hexabromobutadiene (C_4_Br_6_) precursor was prepared using published procedures, as summarized in the Supplementary information; the characterization data match those previously reported [43,44]. Decachloroanthracene (C_14_Cl_10_) precursor was synthesized using procedures in ref. [45].

## CODE AVAILABILITY

The Multiwfn software package is available at http://sobereva.com/multiwfn/.

## Supplementary Material

nwae031_Supplemental_File
