# Supplementary material for: On-surface synthesis and characterization of polyynic carbon chains
Source: Natl Sci Rev. 2024 Jan 22;11(3):nwae031. doi: 10.1093/nsr/nwae031 (PMC10896587; doi:10.1093/nsr/nwae031)
Supplement: nwae031_Supplemental_File [file nwae031_supplemental_file.docx]

Supplementary Information for

**On-surface synthesis** **and characterization of** **polyynic carbon chains**

Wenze Gao, Wei Zheng, Luye Sun, Faming Kang, Zheng Zhou, Wei Xu*

*Corresponding author: xuwei@tongji.edu.cn

**The supplementary information includes:**

Materials and General Methods

Synthetic Protocols

Supplementary Figures 1-11

**Materials and General Methods**

Bruker Ascend 500 (Bruker Biospin, Billerica, MA) spectrometer operating at 500 MHz for 1H and 126 MHz for 13C using tetramethylsilane as the internal standard. Recycling preparative high-performance liquid chromatography (HPLC) was performed with a JAI LC-7080 liquid chromatograph (JAI, Tokyo, Japan) equipped with a JAI UV-800LA UV detector at room temperature. JAIGEL-1HR and JAIGEL-2HR (60 cm × 2.0 cm (i.d.)) connected in series were used as columns (JAI), and chloroform was used as the eluent at a flow rate of 9.0 mL min^–1^.

**Synthetic Protocols**


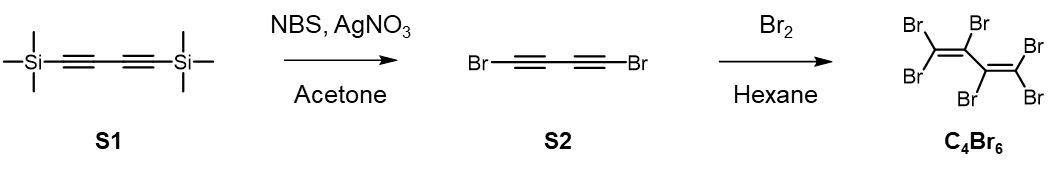


**Supplementary Scheme 1.** Synthesis of **C_4_Br_6_**.

**Synthesis of Dibromobutadiyne (S2)**

To a 500 mL round-bottom flask were added 1,4-bis(trimethylsilyl)-1,3-butadiyne (S1) (0.50 g, 2.58 mmol), Nbromosuccinimide (1.0 g, 5.7 mmol), AgNO_3_ (0.88 g, 5.16 mmol), and acetone (200 mL). The round-bottom flask was wrapped with aluminum foil, and the mixture was stirred at room temperature for 4 h. The solvent was removed in vacuo at 0°C, and the mixture was poured into cooled hexane (200 mL). The solution was washed with ice water, and then dried over Na_2_SO_4_. After removing the solvent by evaporation at 0°C, the crude dibromobutadiyne (400 mg) was obtained as a white flaky solid and was used for the next step without further purification [1]. *Caution: the flaky solid melts and decomposes in seconds at room temperature; therefore, keep it cold and use it immediately after it is prepared.*

**Synthesis of 1,1,2,3,4,4-****hexabromobutadiene (C_4_Br_6_)**

Dibromobutadiyne (0.40 g, 1.92 mmol) was dissolved in cold hexane (4 mL) and the solution was stirred at -25°C. Br_2_ (0.25 mL, 4.8 mmol) was added dropwise with vigorous stirring. After stirring at -25°C for 2 h, the solvent was removed under reduced pressure and the residue was passed through a short pad of silica gel using *n*-hexane/dichloromethane (1/2, v/v) as the eluent. After concentrating in vacuo, the crude product was purified by recycling preparative HPLC on JAIGEL-1H and JAIGEL-2H (60 cm × 2.0 cm (i.d.)) using chloroform as the eluent to give the desired product as a pale yellow solid (600 mg, 59% yield). Mp: 44.8–45.5 °C. ^13^C NMR (126 MHz, CDCl_3_, 25 °C): 121.24, 97.31.


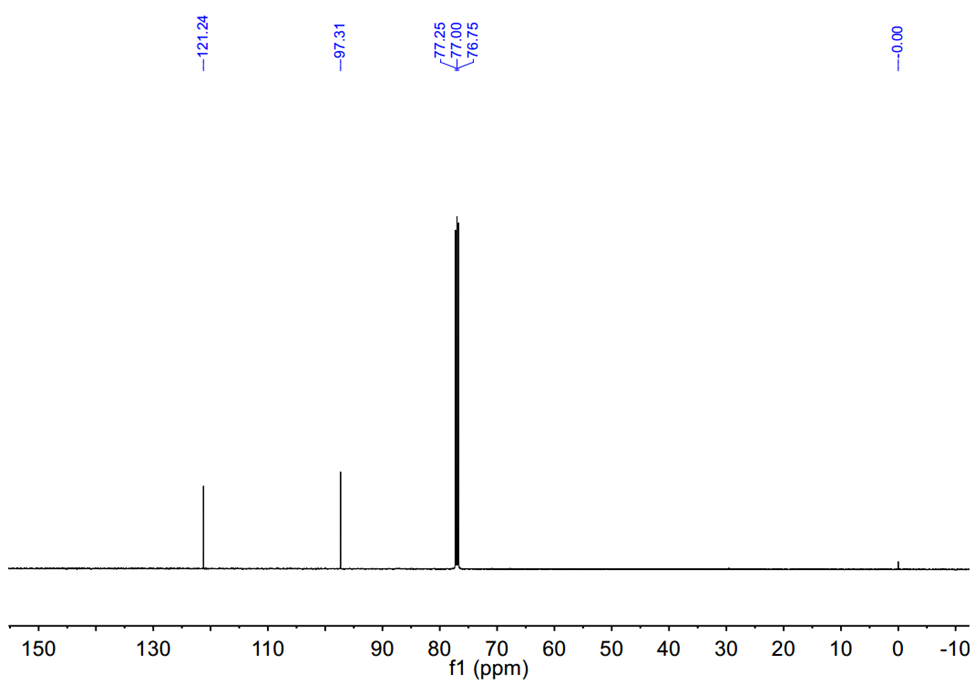


**Supplementary Figure 1.** ^13^C NMR spectrum of **C_4_Br_6_** (126 MHz, CDCl_3_, 25 ℃).


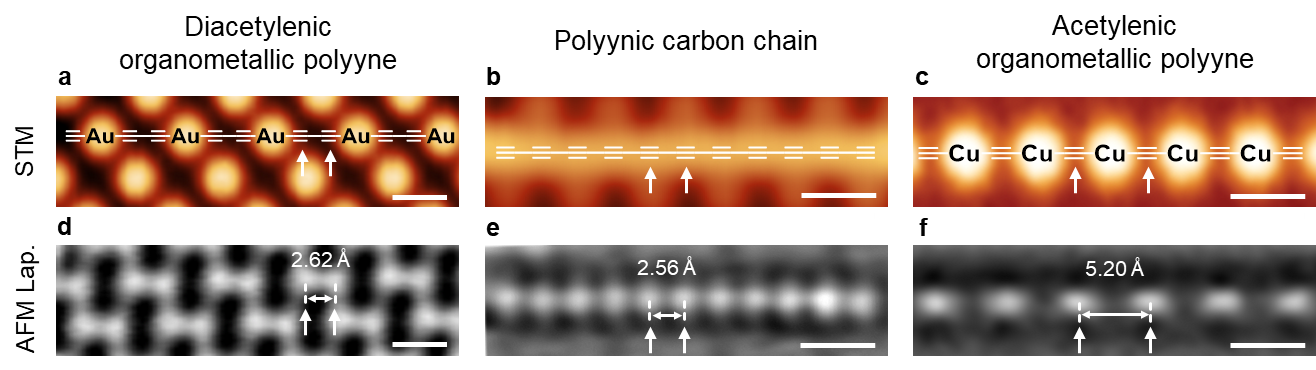


**Supplementary Figure 2. Period comparison between polyynic carbon chain and organometallic polyynes.** STM images (**a**-**c**) and Laplace-filtered AFM images (**d**-**f**) of diacetylenic organometallic polyynes, polyynic carbon chain, and acetylenic organometallic polyyne, respectively. The STM images in **a**-**c** are overlaid with the chemical structures as guides. The STM images are taken at: *V* = 400 mV, *I*_t_ = 100 pA (**a**,**b**); *V* = 5 mV, *I*_t_ = 500 pA (**c**). Scale bars: 0.5 nm.


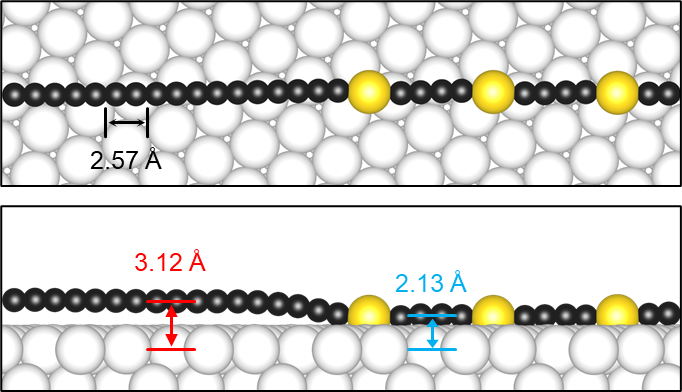


**Supplementary Figure 3.** Top and side-view DFT models of partially demetallized organometallic polyyne on Au(111). Black, gold and white balls represent C, Au atoms in organometallic polyyne, and Au atoms in Au(111) substrate, respectively.


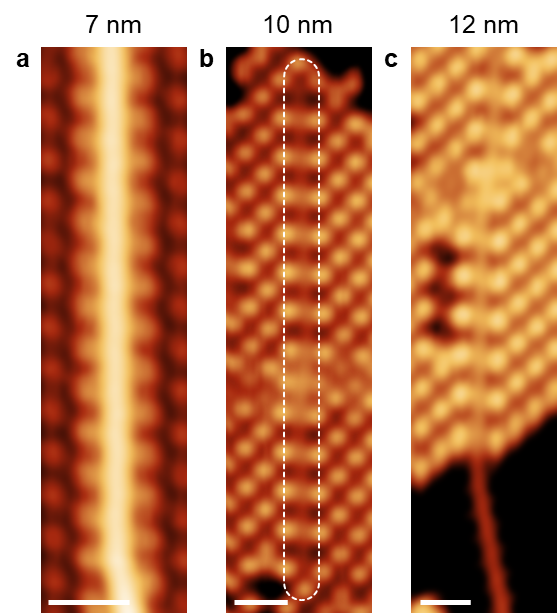


**Supplementary Figure 4.** STM images of other representative long polyynic carbon chains with ~28 (**a**), 40 (**b**) and 48 (**c**) alkyne units, respectively. The STM images are all taken at: *V* = 0.40 V, *I*_t_ = 100 pA. Scale bars: 1 nm.


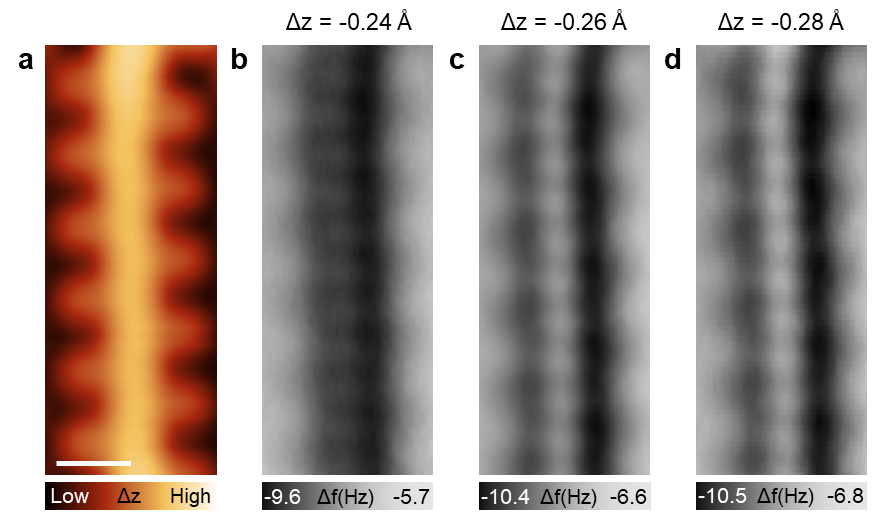


**Supplementary Figure 5**. **AFM measurements of polyyne at different tip heights.** STM image (**a**) and AFM images (**b**-**d**) of the polyynic carbon chain. The AFM images are recorded with a Br-functionalized tip at different tip offsets Δ*z* (b: Δz = -0.24 Å, c: Δz = -0.26 Å, d: Δz = -0.28 Å) with respect to an STM set point (*V* = 0.40 V, *I*_t_ = 100 pA). Scale bars: 0.5 nm. The scale bar in (**a**) applies to all images.


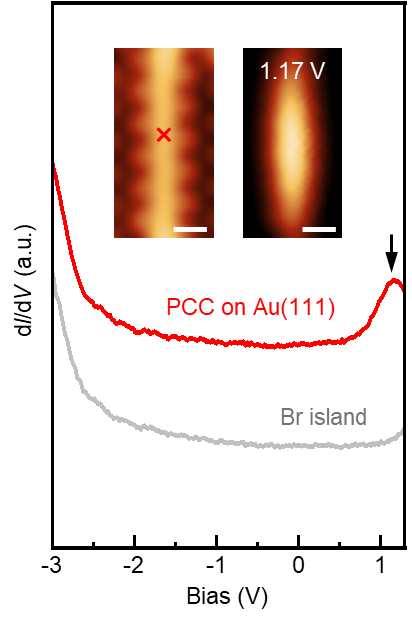


**Supplementary Figure 6.** The d*I*/d*V* spectra of the PCC on Au(111) acquired at the position indicated by the red cross in the inserted STM image (red curve) and the reference spectrum taken on the Br island (grey curve). The inserted constant-height d*I*/d*V* map is acquired at 1.17 V.


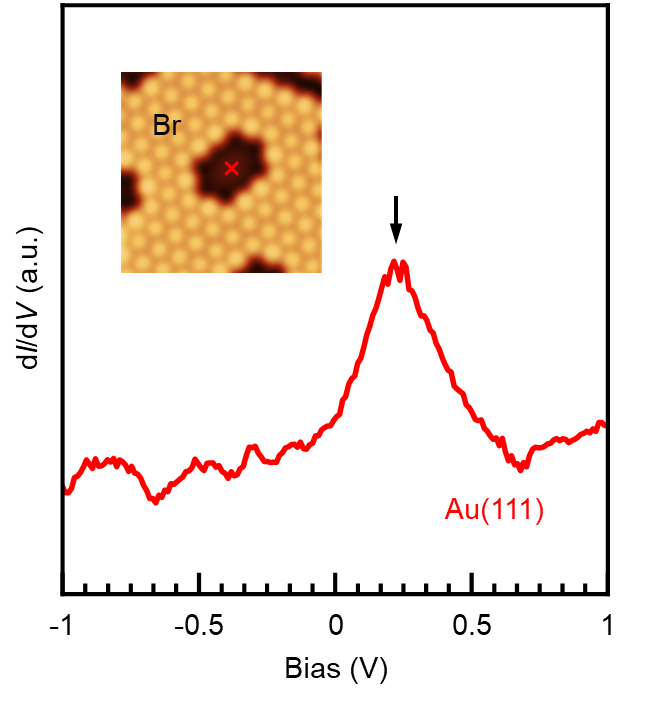


**Supplementary Figure 7.** The d*I*/d*V* spectrum of the Au(111) surface acquired at the position indicated by the red cross in the inserted STM image.


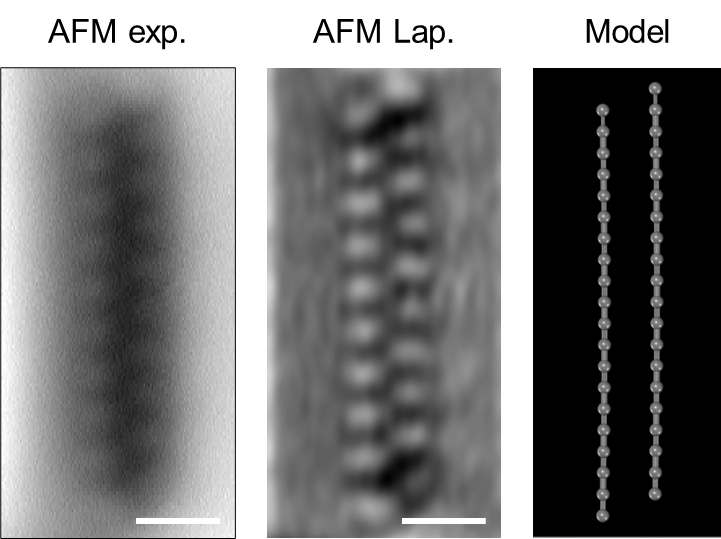


**Supplementary Figure 8.** The close-up AFM image, corresponding Laplace-filtered AFM image, and the schematic model of decoupled PCCs shown in manuscript Fig. 4c. The AFM image is recorded with a Br-functionalized tip at tip offset Δ*z* = -160 pm with respect to an STM set point (*V* = 0.40 V, *I*_t_ = 100 pA). Scale bars: 0.5 nm.

**Supplementary Figure 9.** Constant-height dI/dV maps of partially decoupled PCC acquired at 0.87 V (**a**) and -2.8 V (**d**), respectively. **b**, Corresponding Laplace-filtered image of (**a**). DFT-orbital maps at the LUMO (**c**) and HOMO (**e**) resonances PCC with 10 alkyne units. Scale bars: 0.5 nm.

**Supplementary Figure 10**. DFT-calculated frontier molecular orbitals of C_14_ and C_14_H_2_ chains. The DFT calculations were carried out at the ωB97XD/def2-TZVP level. Green and blue isosurfaces represent the positive and negative phase values of electron orbitals, respectively. Isovalue for molecular orbitals is 0.004.


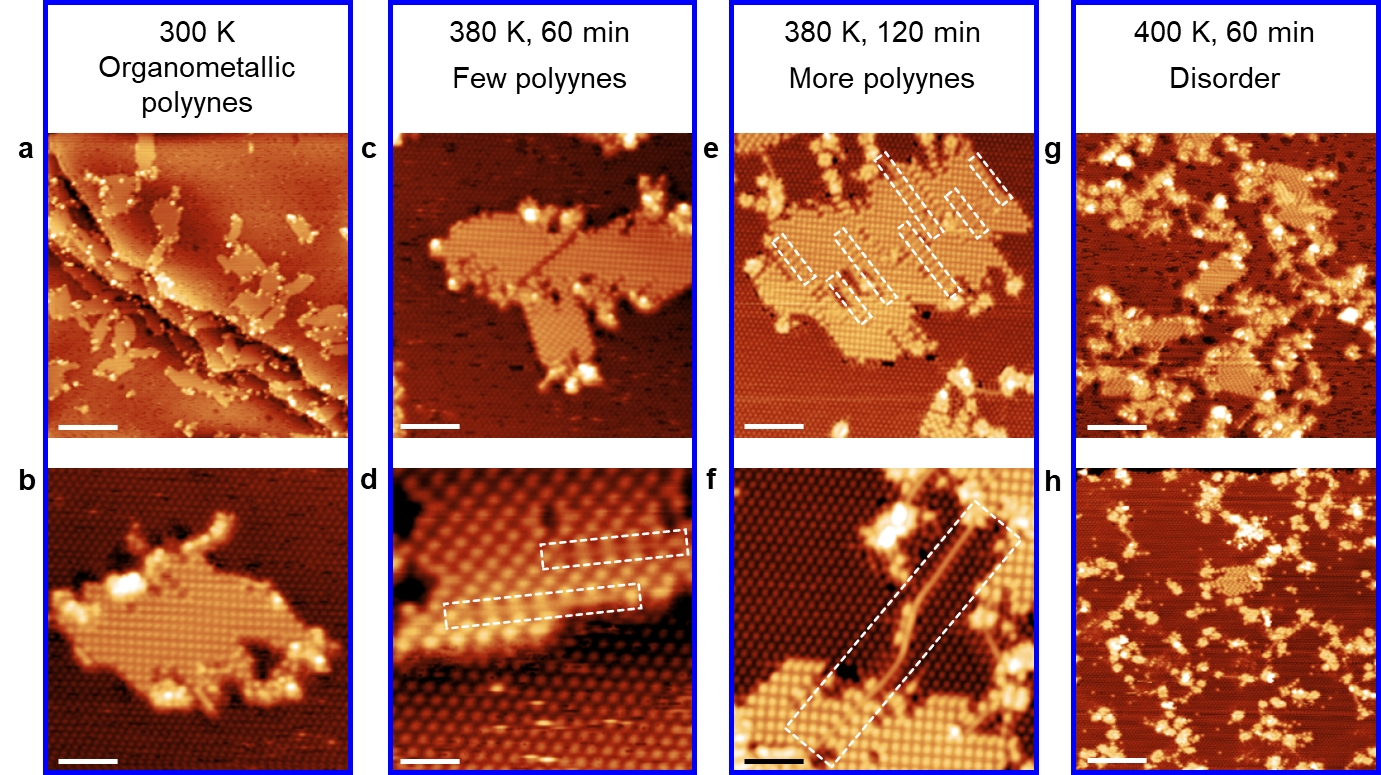


**Supplementary Figure 11.** STM overview images of the surface after the different annealing procedures. Polyynic carbon chains are outlined by white contours. The STM images are all taken at: *V* = 0.40 V, *I*_t_ = 100 pA. Scale bars: 20 nm (**a**); 4 nm (**b**); 6 nm (**c**,**e**); 2 nm (**d**); 3 nm (**f**); 10 nm (**g**,**h**).

**Reference**

1. Dembinski R, Bartik T, Bartik B *et al*. Toward metal-capped one-dimensional carbon allotropes: wirelike C_6_−C_20_ polyynediyl chains that span two redox-active (η^5^-C_5_Me_5_)Re(NO)(PPh_3_) endgroups. *J Am Chem Soc* 2000; **122**: 810-22.
